# Insulin-like growth factor-1 effects on kidney development in preterm piglets

**DOI:** 10.1038/s41390-024-03222-3

**Published:** 2024-05-18

**Authors:** Jingren Zhong, Richard Doughty, Thomas Thymann, Per Torp Sangild, Duc Ninh Nguyen, Tik Muk

**Affiliations:** 1 Section for Comparative Paediatrics and Nutrition, Department of Veterinary and Animal Sciences, University of Copenhagen, Frederiksberg, Denmark; 2 Department of Pathology, Akershus University Hospital, Lørenskog, Norway; 3 Department of Pediatrics, Odense University Hospital, Odense, Denmark; 4 Department of Neonatology, Rigshospitalet, Copenhagen, Denmark

## Abstract

**Background:**

Preterm birth disrupts fetal kidney development, potentially leading to postnatal acute kidney injury. Preterm infants are deficient in insulin-like growth factor 1 (IGF-1), a growth factor that stimulates organ development. By utilizing a preterm pig model, this study investigated whether IGF-1 supplementation enhances preterm kidney maturation.

**Methods:**

Cesarean-delivered preterm pigs were treated systemically IGF-1 or vehicle control for 5, 9 or 19 days after birth. Blood, urine, and kidney tissue were collected for biochemical, histological and gene expression analyses. Age-matched term-born pigs were sacrificed at similar postnatal ages and served as the reference group.

**Results:**

Compared with term pigs, preterm pigs exhibited impaired kidney maturation, as indicated by analyses of renal morphology, histopathology, and inflammatory and injury markers. Supplementation with IGF-1 reduced signs of kidney immaturity, particularly in the first week of life, as indicated by improved morphology, upregulated expression of key developmental genes, reduced severity and incidence of microscopic lesions, and decreased levels of inflammatory and injury markers. No association was seen between the symptoms of necrotizing enterocolitis and kidney defects.

**Conclusion:**

Preterm birth in pigs impairs kidney maturation and exogenous IGF-1 treatment partially reverses this impairment. Early IGF-1 supplementation could support the development of preterm kidneys.

**Impact:**

- Preterm birth may disrupt kidney development in newborns, potentially leading to morphological changes, injury, and inflammation.
- Preterm pigs have previously been used as models for preterm infants, but not for kidney development.
- IGF-1 supplementation promotes kidney maturation and alleviates renal impairments in the first week of life in preterm pigs. IGF-1 may hold potential as a supportive therapy for preterm infants sensitive to acute kidney injury.

## Introduction

In humans, nephrogenesis is completed by around 36 weeks gestation, with approximately 60% of nephrons formed during the last trimester of gestation at the time when preterm infants are delivered.^1^ Preterm birth, which induces a series of whole-body complications,^2,3^ may alter fetal renal development, compromise nephrogenesis, and increase the risk of renal dysfunction, insufficiency, and failure.^4,5^ Preterm infants exhibit impaired glomerular and tubular function,^6–8^ and show an increased vulnerability to acute kidney injury (AKI) during the postnatal period.^9,10^ The kidney develops through branching morphogenesis during the pre- and postnatal period, which involves ureteric bud elongation and mesenchymal-epithelial transformation.^11–13^ These processes are regulated by a set of signaling pathways, including the Wnt signaling pathway and the glial cell line-derived neurotrophic factor (GDNF)/Ret proto-oncogene (RET) signaling pathway.^14–16^ Up to date, there is limited knowledge of how preterm birth, and its associated complications may impact renal development and related signaling pathways.

Besides, as nephrogenesis is sensitive to inflammatory insults,^17,18^ impaired renal development may result not only from immaturity but also from inflammation-related complications after preterm birth. Necrotizing enterocolitis (NEC) is an inflammatory disorder of gastrointestinal tract that mostly affects premature infants. NEC can range from mild mucosal lesions to severe mucosal destruction, often accompanied by systemic inflammation and sepsis.^19–21^ Severe NEC lesions, coupled with systemic inflammation, may impact distant organs such as the kidney and exacerbate the risk of AKI.^20,22,23^ However, the impact of milder forms of neonatal gastrointestinal lesions on kidney health remains unknown.

Despite its clinical importance, there are limited options available for preventing or treating prematurity-related AKI and its clinical complications. Insulin-like growth factor 1 (IGF-1) is a crucial growth factor with a range of mitogenic, differentiating, and metabolic effects throughout the body during both fetal and postnatal life.^24^ IGF-1 is expressed in all fetal tissues and exhibits autocrine, endocrine, and paracrine actions mediated via both IGF-1 and insulin receptors.^25^ IGFs circulate in plasma and form complexes with a family of structurally related IGF-binding proteins. Approximately 80% of IGF-1 in circulation is bound to IGF-binding protein 3 (IGFBP-3) via an acid-labile subunit.^26^ This complex plays a pivotal role in regulating the action and biological availability of IGF-1 in peripheral tissues.^26^ In preterm infants, circulating IGF-1 levels are considerably lower than those of their counterparts in utero. This reduction may contribute to developmental deficiencies after birth, including kidney immaturity.^27–29^ Previous studies have demonstrated that exogenous administration of IGF-1 increases kidney weight in fetuses^30^ and stimulates renal filtration and reabsorption.^28^ However, IGF-1 can also play a role in kidney fibrosis and inflammation.^28,31^ The above evidence underscores the need for further investigations to determine both the safety and any potential beneficial effects of IGF-1 supplementation on preterm kidneys.

Based on our previous studies investigating the effects of IGF-1 supplementation on the gut development and NEC,^32–34^ we explored whether IGF-1 supplementation affects kidney development in preterm pigs, serving as a model for preterm infants. Pigs delivered at 90% gestation may exhibit notable signs of kidney immaturity, as nephrogenesis in pigs continues until postnatal week three.^13^ This immaturity renders preterm pigs highly susceptible to extrauterine insults, similar to the vulnerability observed in preterm infants delivered at earlier (approximately 70%) gestation. Using preterm pigs as a model, we hypothesized that preterm birth is associated with disrupted nephrogenesis and renal structure formation, and that postnatal supplementation with IGF-1 promotes kidney maturation and mitigates the impacts of preterm birth on kidney structure.

## Methods

### Animal model and necrotizing enterocolitis evaluation

**Fig. 1:**
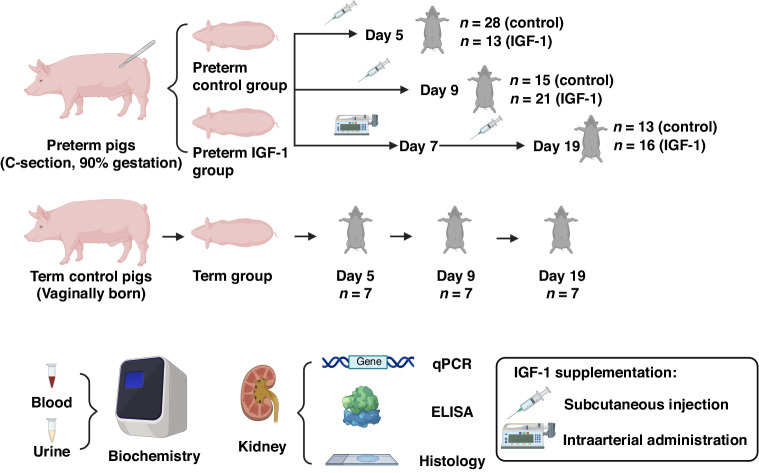
Study overview. Throughout the three experiments, preterm pigs were treated with either vehicle or rhIGF-1/IGFBP-3 for 5, 9 and 19 days. Vaginally born, farm-reared term pigs were euthanized at matching postnatal ages and served as the reference group. After euthanasia, kidney tissue, blood and urine samples were collected for further analysis.

Animal studies were conducted in accordance with the European Communities Council Directive 2010/63/EU and approved by the Danish Animal Experiments Inspectorate. The study design, summarized in Fig. 1, contains three experimental groups: the preterm control group, the preterm IGF-1 group and the term reference group. The current study utilized available kidney samples that were obtained from our previous studies, which focused on recombinant human IGF-1 with binding protein 3 (rhIGF-1/IGFBP-3) supplementation in preterm pigs for the prevention of NEC.^32–34^ These prior investigations have reported some basic characteristics data of animal experiments and revealed that supplementation of IGF-1 moderately improved NEC resistance and overall survival after a treatment period of 5-19 days.

Preterm pigs were delivered by cesarean section at 90% gestation from seven sows (Landrace x Yorkshire x Duroc, 106 days, two litters for the 5-d study, three litters for the 9-d study, and two litters for the 19-d study). Preterm pigs were block randomized based on their birth weight and sex. The pigs were then divided into two groups: the preterm IGF-1 group, which received rhIGF-1/IGFBP-3; and the preterm control group, which received equivalent volumes of a vehicle solution. Since IGF-1 shares an identical amino acid sequence in humans and pigs,^35^ it is anticipated that rhIGF-1 will exert a similar effect when binding to the IGF-1 receptor in pigs. The rhIGF-1/IGFBP-3 complex was administered by subcutaneous injection at a dose of 0.25 mg/kg per injection, twice daily during the 5-d study and three times daily during the 9-d study, as described previously.^32,33^ During the 19-d study, rhIGF-1/IGFBP-3 was administered via intra-arterial infusion through umbilical artery catheters for the first 7 days at a dose of 2.25 mg/kg/day. After catheter withdrawal on postnatal day 8 (PND8), administration was switched to subcutaneous injections, three times daily at a dose of 0.25 mg/kg per injection.^34^ Pigs were immunized and nourished with parenteral nutrition and infant formula, as described previously.^33^ Reference pigs (Landrace x Yorkshire x Duroc, 117 ± 2 days, 3 litters) born vaginally at term were sow-reared until euthanasia. At the designated endpoints of the experiment, piglets were anaesthetized with an intramuscular injection of 0.1 mL/kg Zoletile-pigmix (50 mg/mL), followed by euthanasia using pentobarbital (Euthanimal, 400 mg/mL, 0.25 ml/kg) administered via heart puncture. In the current study, only pigs that survived until scheduled euthanasia were included in tissue sampling and subsequent laboratory analyses. Blood, urine and kidney tissue from term and preterm pigs were collected on identical postnatal days (PND5, PND9, or PND19) to ensure matching postnatal ages between preterm and term groups. For tissue collection, kidneys were cut longitudinally to include the renal capsule, cortex, medulla and pelvis. One half was fixed in phosphate buffer paraformaldehyde for histology, and the other half was snap-frozen in liquid nitrogen and stored at −80 °C for further analysis (gene and protein expression). Blood was collected with a heparin vacutainer (BD Diagnostics, Oxford, UK) via heart puncture after anesthesia and urine was collected via cystopuncture after euthanasia. All available kidney, blood and urine samples were included in the analysis. The blood IGF-1 levels of the pigs included in the present study are shown in Table 1. The detailed rhIGF-1/IGFBP-3 pharmacokinetics in piglets were reported previously.^32,33^

**Table 1:** Body and kidney weights, and circulating IGF-1 levels at the time of euthanasia in term and preterm pigs supplemented with/without IGF-1^a^.

			Group			*p*-value	
			Preterm control	Preterm IGF-1	Term	Preterm control vs. Preterm IGF-1	Preterm control vs. Term
PND5			*n* = 28	*n* = 13	*n* = 7		
Birth weight (g)			957.9 ± 54.0	1038.3 ± 65.9	-	0.62	-
Euthanasia weight (g)			1008.4 ± 67.5	1092.4 ± 69.1	1688.1 ± 69.9	0.86	<0.001
Kidney weight (g)			9.3 ± 0.6	9.7 ± 0.8	13.7 ± 0.8	0.87	<0.001
Circulating IGF-1 (ng/ml)			11.2 ± 1.2	49.8 ± 4.9	16.1 ± 2.9	<0.001	<0.05
PND9			*n* = 15	*n* = 21	*n* = 7		
Birth weight (g)			947.9 ± 74.1	953.8 ± 54.4	-	0.80	-
Euthanasia weight (g)			1238.8 ± 86.0	1269.8 ± 84.4	3229.4 ± 244.6	0.97	<0.001
Kidney weight (g)			11.9 ± 1.0	13.5 ± 1.2	27.3 ± 2.2	0.68	<0.001
Circulating IGF-1 (ng/ml)			18.7 ± 2.7	87.6 ± 11.7	46.9 ± 7.2	<0.001	<0.01
PND19			*n* = 13	*n* = 16	*n* = 7		
Birth weight (g)			995.7 ± 50.0	903.0 ± 48.4	-	0.17	-
Euthanasia weight (g)			1976.6 ± 70.3	1790.3 ± 74.9	4491.4 ± 457.6	0.60	<0.001
Kidney weight (g)			14.0 ± 0.8	14.5 ± 0.8	33.0 ± 3.1	0.93	<0.001
Circulating IGF-1 (ng/ml)			42.9 ± 2.5	139.3 ± 9.8	45.4 ± 8.6	< 0.001	0.57

^a^Data are presented as mean ± SEM.

After euthanasia, macroscopic indicators of NEC were assessed by two independent observers, based on the presence of signs in the stomach; proximal, middle, and distal small intestine; and colon, as previously described.^36–38^ Briefly, lesion severity for each of the gut regions was evaluated using a validated NEC scoring system as follows: 1 = absence of lesions, 2 = local hyperaemia, 3 = hyperaemia, extensive edema and local hemorrhage, 4 = extensive hemorrhage, 5 = local necrosis or pneumatosis intestinalis, 6 = extensive necrosis and pneumatosis intestinalis. To further examine the effect of NEC on the kidney, preterm pigs euthanized on PND5 were subdivided into three groups according to NEC severity: no NEC (all scores = 1), mild NEC (highest score in all gut regions = 2–3), and severe NEC (highest score in all gut regions = 4–6). Furthermore, all preterm pigs in the 5-d study were subdivided into three groups according to lesion type: no lesion (all scores ≤ 2), small intestinal (SI) lesion (lesion score from SI ≥ 3), and non-SI tract lesion (lesion score from stomach and colon ≥ 3, and lesion score from SI ≤ 2). Kidney parameters were compared among the groups to determine the influence of NEC severity and lesion type on renal health.

### Biochemical analysis

The plasma and urine biochemical parameters were analyzed using an Advia 1800 Chemistry System (Siemens Healthcare, Ballerup, Denmark). Conventional markers for kidney function assessment were included in the biochemistry profiling. Additionally, some of these biochemical data were reported in previous studies on NEC and gut development outcomes.^32–34^ Estimated glomerular filtration rate (eGFR) was calculated based on a formula established in pigs using plasma creatinine levels.^39^

### Histomorphological evaluation and morphometry

Formalin fixed kidney tissues were embedded in paraffin, cut into slices of 4 μm, and stained with hematoxylin and eosin (HE) and periodic acid–Schiff (PAS) stain. The kidney slices were evaluated and interpreted by a pathologist without prior knowledge of the treatment groups. Renal pathology was classified according to changes in the glomeruli, tubules and interstitium, and graded on a 4-point scale (none; mild; moderate and marked). The nephrogenic zone (NGZ) was defined as the region in the outer renal cortex containing developing glomerular structures in the form of comma and S-shaped bodies and appearing as a blue strip in HE sections. The width of the NGZ represents the residual nephrogenic potential of each neonatal kidney and was previously used to assess renal maturity in neonates of a variety of species.^40–42^ The nephrogenic zone width (NZW) was measured in five randomly selected regions of the cortex using HE sections, and the average width per kidney was calculated.^43^ Mature glomeruli were counted along five well-defined medullary rays in each kidney sample, and the average number of glomerular generations per kidney was determined. The number of glomerular generations reflects renal maturity and nephron endowment.^44,45^ Glomerular area and density were assessed using PAS sections.^46–48^ Glomerular areas were calculated by measuring five randomly identified glomeruli with clear capillary tufts and Bowman’s space in each field of view. The cross-sectional glomerular density was determined by counting the number of glomeruli in one central transverse cross-section of the kidney at low-power magnification (20×), and then dividing by the total cortex area of the same section. Abnormal glomeruli with shrunken tufts were counted together with all glomeruli in the field to calculate the percentage of abnormal glomeruli.^41,49^ Since injury to the vasculature of the kidney is closely related to the deposition of PAS-positive extracellular matrix,^50^ the fractional mesangial area (FMA) was adopted to quantify the percentage of PAS-positive mesangial matrix within the glomerular tuft to reflect injury in the glomeruli (400× magnification).^51^ The FMA method is recommended by the Diabetes Complications Consortium protocols for evaluating kidney injury, and was previously utilized to assess premature-related kidney glomerular vasculature injury in our neonatal piglet model.^52^ Further experiments might be necessary to thoroughly validate this method for use in neonatal research. All morphometric analyses were performed using ImageJ software version 1.50i (NIH, Bethesda, MD).

### RT-qPCR and enzyme-linked immunosorbent assay

Gene expression levels in the renal cortex were determined by RT-qPCR. The primers used are shown in Supplemental Table S1. Briefly, total RNA was extracted from homogenized kidney cortex by using RNeasy Mini Kit (Qiagen, Copenhagen, Denmark) according to manufacturer’s instructions. RNA was reverse transcribed into cDNA using High-Capacity cDNA Reverse Transcription Kit (Thermo Fisher Scientific, United States). RT-qPCR was subsequently performed using the QuantiTect SYBR Green PCR Kit (Qiagen) on the LightCycler 480 (Roche, Hvidovre, Denmark), and expression levels of the target genes were normalized to the housekeeping gene hypoxanthine phosphoribosyltransferase 1 (*HPRT1*).^53^ Renal tumor necrosis factor alpha (TNFα) and interleukin 10 (IL10) protein levels were measured by enzyme-linked immunosorbent assay (ELISA) kits (R&D Systems, Abingdon, UK).

### Data analysis and statistics

Univariate analysis was applied to all RT-qPCR, ELISA, biochemical and morphometrical data at different time points of sampling using R Studio 3.6.1 (R Studio, Boston, MA, United States). Each parameter was fitted to a linear mixed-effects model with group and sex as the fixed factors, and litter as the random factor using the lme4 package. The data were transformed if the original data did not fit properly into the model after checking the residuals. Binary data was analyzed using a logistic regression model. The NEC score, treated as an ordered categorical outcome, was analyzed by a proportional odds logistic regression model. Histomorphological incidences were analyzed using Fisher’s exact test. A *p*-value < 0.05 was regarded as statistically significant. The data are presented as mean ± SEM.

## Results

### Clinical outcomes

An overview of the clinical parameters is shown in Table 1. Term pigs had significantly higher body weight and kidney weight compared to preterm pigs at all PNDs at the time of euthanasia (*p* < 0.001). Body weight and kidney weight were similar between the preterm control and preterm IGF-1 groups. The incidence and distribution of NEC lesions in preterm pigs involved in the current study are shown in Supplemental Fig. S1a–c. The preterm IGF-1 group showed a lower incidence of NEC than the preterm control group on PND5 (*p* = 0.08). However, the lesion severity across all regions was mild and similar between the groups. Across groups, the severity or type of NEC lesions had minimal effects on the observed kidney-related parameters. Notably, only pigs with SI lesions demonstrated a significant higher relative kidney weight compared to pigs with no digestive tract lesions (*p* < 0.01, Supplemental Fig. S2 and S3).

### Effects of IGF-1 treatment on kidney development following preterm birth

**Fig. 2:**
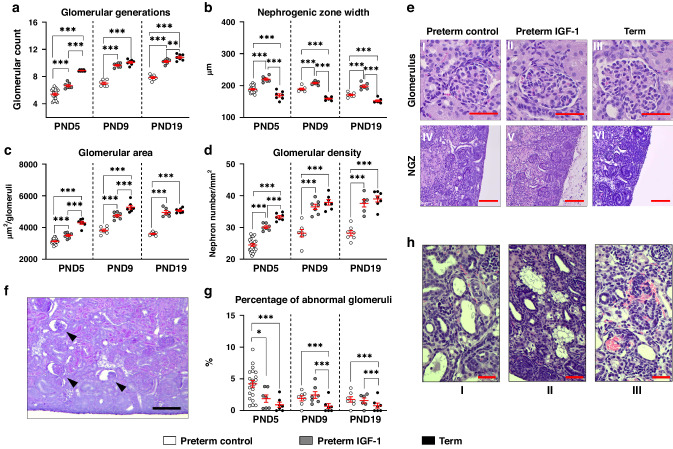
Effects of preterm birth-associated immaturity and IGF-1 supplementation on kidney structure and maturation during the first 19 postnatal days. **a**–**d** Morphometry data evaluated from kidney sections. Quantitative analysis of (**a**) medullary ray glomerular generation; (**b**) the width of the nephrogenic zone; (**c**) the cross-sectional area of the renal corpuscle; (**d**) glomerular density. **e** Representative histological images showing the glomerulus (I-III, HE, 600×, scale bar: 100 µm) and nephrogenic zone (NGZ, IV-VI, HE, 100×, scale bar: 200 µm) of each group on PND5. **f** Representative histological images of the preterm control group on PND5, indicating abnormal glomeruli (cystic dilation of Bowman’s space and shrunken glomerular tuft) in the outer renal cortex (HE, 100×, scale bar: 200 µm). **g** Percentage of abnormal glomeruli. **h** Representative histological images showing pathologies with a higher incidence in the preterm control group than the preterm IGF-1 group on PND9. I: Tubule dilatation; II: Vacuolation of the proximal tubule; III: Interstitial and glomerular hemorrhage (HE, 400×, scale bar: 50 µm). All the data for preterm control (*n* = 7–36), preterm IGF-1 (*n* = 7–24) and term reference pigs (*n* = 6–7) are presented as the means ± SEM. *p < 0.05, **p < 0.01, ***p < 0.001.

Overall, there was no significant difference in relative kidney weight between preterm and term animals. However, IGF-1 supplementation increased the relative kidney weight of preterm pigs on PND19 (*p* < 0.05, Supplemental Fig. S1d). The histological assessment of kidney maturation and structure is shown in Fig. 2. Compared to term pigs at the same gestational age, preterm control pigs exhibited significantly lower glomerular generation numbers, areas, and densities, as well as significantly greater NZW (all *p* < 0.001, Fig. 2a–e). IGF-1 supplementation significantly increased glomerular generation numbers, areas, densities, and NZW in preterm animals at all time points (all *p* < 0.001, Fig. 2a–e), bringing these values (except for NZW) close to those in term reference pigs. Notably, no significant differences were observed between the preterm IGF-1 group and the term group when assessing glomerular generation numbers on PND9, glomerular areas on PND19, and glomerular densities on both PND9 and PND19. This indicates improved maturation of preterm kidneys after IGF-1 supplementation. Abnormal glomeruli were characterized by cystic dilation of Bowman’s space and shrunken glomerular tufts in the superficial renal cortex^4,41,54^ (Fig. 2f). The percentage of abnormal glomeruli was significantly greater in the preterm control group on PND5 than in the preterm IGF-1 (*p* < 0.05) and term reference groups (*p* < 0.001). On PND9 and PND19, both preterm groups exhibited a higher percentage of abnormal glomeruli compared to the term group (*p* < 0.001, Fig. 2g). The morphological alterations of the kidney included tubular dilatation and vacuolization, interstitial edema and hemorrhage, glomerular capillary hemorrhage, and cystic dilatation of Bowman’s space. The incidence and severity of these morphological changes were increased in preterm control pigs compared to term pigs from PND5 to 19. Interestingly, compared with preterm control pigs, IGF-1 supplementation reduced the incidence and severity of renal lesions, especially on PND9 and 19 (Table 2 and Fig. 2h).

**Table 2:** Effects of preterm birth associated immaturity and IGF-1 supplementation on the frequency of kidney histological pathologies (%) during the first 19 postnatal days ^a^.

Histological pathology		Group		
PND5		Preterm control	Preterm IGF-1	Term
		*n* = 22	*n* = 7	*n* = 7
**Glomeruli**				
Cystic dilatation ^b^		40.9	28.6	14.3
Hemorrhage		31.8	28.6	0
**Tubules**				
Dilatation ^c^		50.0	28.6	14.3
Mild		36.4	28.6	14.3
Moderate		9.1	0	0
Marked		4.5	0	0
Proximal tubule vacuolization		31.8	14.3	0
Mild		9.1	14.3	0
Moderate		13.6	0	0
Marked		9.1	0	0
**Interstitium**				
Edema		36.4	28.6	0
Hemorrhage		36.4	28.6	14.3
**PND9**		**Preterm control**	**Preterm IGF-1**	**Term**
		*n* = 7	*n* = 7	*n* = 7
**Glomeruli**				
Cystic dilatation		28.6	0	0
Hemorrhage		28.6	0	0
**Tubules**				
Dilatation		100.0 ^a^	42.9 ^ab^	14.3 ^b^
Proximal vacuolization		42.9	0	0
**Interstitium**				
Edema		42.9	0	0
Hemorrhage		14.3	0	0
**PND19**		**Preterm control**	**Preterm IGF-1**	**Term**
		*n* = 7	*n* = 6	*n* = 7
**Glomeruli**				
Cystic dilatation		14.3	0	0
Hemorrhage		0	0	0
**Tubules**				
Dilatation		71.4 ^a^	16.7 ^ab^	0 ^b^
Proximal vacuolization		28.6	0	0
**Interstitium**				
Edema		28.6	0	0
Hemorrhage		0	0	14.3

^**a**^The histological pathologies were based on microscopy evaluation by pathologists. The degree of histological pathologies was assessed to four levels: none, mild, moderate and marked. Within a row, different superscript letters indicate statistical significance (*p*-value < 0.05).

^**b**^The pathology was mild if no degrees of the pathology were mentioned.

^**c**^The total frequency of the pathology of any degrees.

**Fig. 3:**
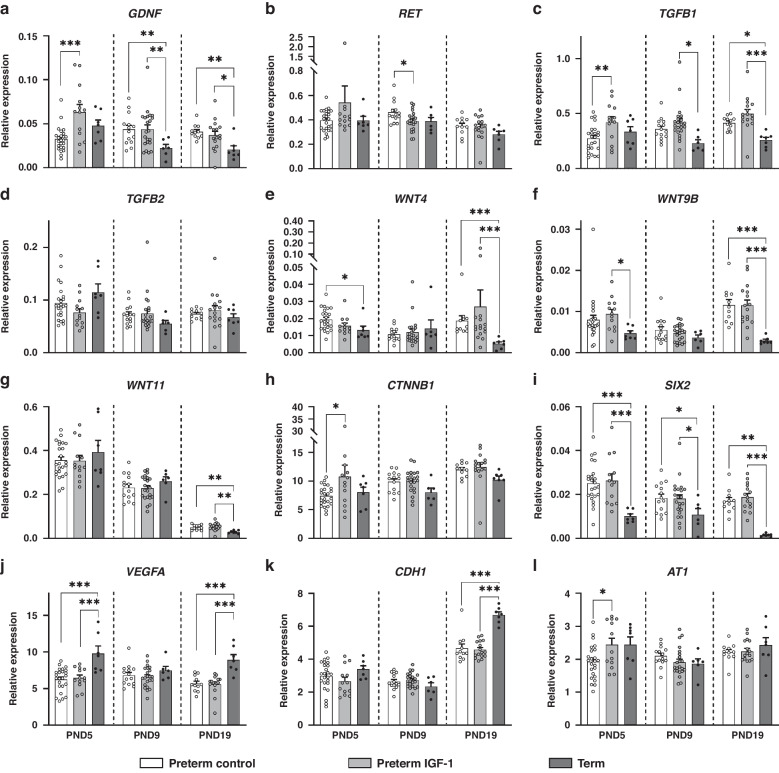
Effects of preterm birth-associated immaturity and IGF-1 supplementation on kidney development-related gene expression during the first 19 postnatal days. Relative gene expression of (**a**, **b**) Glial cell line-derived neurotrophic factor/Ret proto-oncogene signaling molecules (*GDNF* and *RET*), (**c**, **d**) transforming growth factor-beta (*TGFB1* and *TGFB2*), (**e**–**h**) Wnt family members and their downstream molecule β-catenin *(WNT4*, *WNT9B*, *WNT11* and *CTNNB1*), (**i**) the nephron progenitor marker *SIX2*, and (**j**–**l**) other kidney development-related molecules (*VEGFA*, *CDH1*, and *AT1*) in kidney tissue. All the data for the preterm control (*n* = 11–24), preterm IGF-1 (*n* = 12–22) and term reference pigs (*n* = 6–7) are presented as the means ± SEM. *p < 0.05, **p < 0.01, ***p < 0.001.

The expression of renal development-related genes is shown in Fig. 3. Preterm pigs displayed a different gene expression pattern related to kidney development compared to term pigs, as evidenced by the distinct expression of SIX homeobox 2 (*SIX2*), vascular endothelial growth factor-A (*VEGFA*), *GDNF*, transforming growth factor beta 1 (*TGFB1*), Wnt family member 4 (*WNT4*), *WNT9B*, *WNT11*, and E-cadherin (*CDH1*). Furthermore, IGF-1 supplementation significantly upregulated the expression of *GDNF*, *TGFB1*, angiotensin II type 1 receptor (*AT1*) and catenin beta 1 (*CTNNB1*) on PND5 in preterm animals (all *p* < 0.05). However, no differences in gene expression were detected on PND9 or PND19 between the preterm IGF-1 group and the preterm control group. These findings provide evidence for the promotive effect of IGF-1 supplementation on early-stage kidney maturation after preterm birth.

### Effects of IGF-1 treatment on kidney function and injury

Kidney function and injury status were initially analyzed by examining plasma and urine biochemistry as illustrated in Supplemental Fig. S4. On PND5, no differences in plasma creatinine levels were found among the groups, while on PND9 and 19, term pigs had relatively higher plasma creatinine levels, compared to preterm born pigs. Moreover, the blood urea nitrogen (BUN) level in term pigs was higher than in preterm pigs on PND5 and PND19 (*p* < 0.01 and *p* < 0.05, respectively). The plasma albumin concentration was consistently higher in term pigs than in preterm pigs (*p* < 0.05). Higher urine albumin/creatinine levels were found in the term vs. preterm IGF-1 group on PND9 only (*p* < 0.05). All the measured biochemical parameters and eGFR showed no significant difference between the preterm control and IGF-1 pigs.

**Fig. 4:**
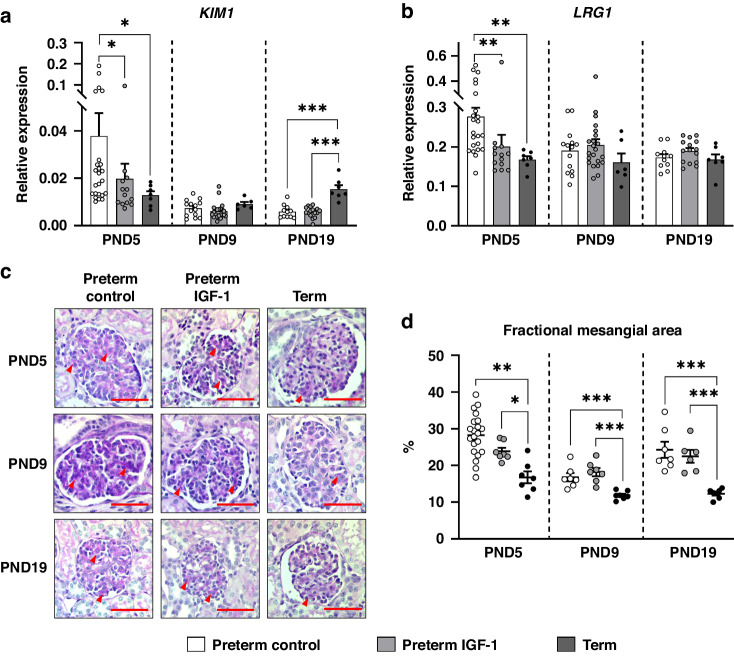
Effects of preterm birth-associated immaturity and IGF-1 supplementation on kidney injury-related parameters during the first 19 postnatal days. **a**, **b** Relative gene expression of kidney injury markers (*KIM1* and *LRG1*) in renal tissue. **c** Representative histological images of the glomeruli in the preterm control, preterm IGF-1 and term groups (PAS, ×600, scale bar: 50 µm). Arrowheads indicate the extracellular matrix in the glomerular vasculature strongly stained by PAS. **d** Fractional mesangial area of the glomeruli. All data for the preterm control (*n* = 7–21), preterm IGF-1 (*n* = 7–22) and term reference pigs (*n* = 6–7) are presented as the means ± SEM. *p < 0.05, **p < 0.01, ***p < 0.001.

To assess kidney injury, the expression of kidney injury-related genes was measured, as shown in Fig. 4. The expression of kidney injury molecule-1 (*KIM1, p* < 0.05) and leucine-rich alpha-2-glycoprotein 1 (*LRG1, p* < 0.01) was significantly higher in the preterm control group than in the preterm IGF-1 and term groups on PND5 (Fig. 4a, b), suggesting potential protective effects of IGF-1 against preterm birth-related kidney injury. At all time points, FMA, which indicates the degree of glomerular injury and fibrosis, was higher in the preterm control and IGF-1 groups than in the term group (*p* < 0.05, Fig. 4c, d).

### Effects of IGF-1 treatment on kidney inflammation-related genes and proteins

**Fig. 5:**
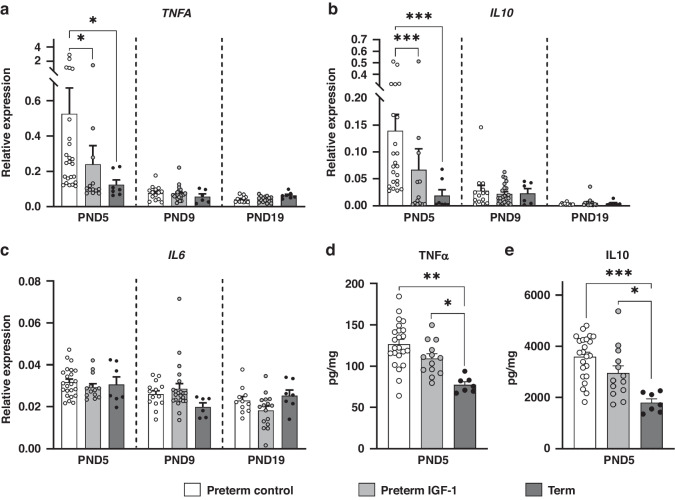
Effects of preterm birth-associated immaturity and IGF-1 supplementation on kidney inflammation-related parameters during the first 19 postnatal days. **a**–**c** Relative gene expression of three inflammatory cytokines (*TNFA*, *IL10* and *IL6*) in kidney tissue. **d**, **e** Renal protein expression of two inflammatory cytokines (TNFα and IL10). All data for the preterm control (*n* = 11–24), preterm IGF-1 (*n* = 12–22) and term reference pigs (*n* = 6–7) are presented as the means ± SEM. *p < 0.05, **p < 0.01, ***p < 0.001.

As shown in Fig. 5, preterm birth was associated with increased inflammatory responses in the kidney on PND5, as indicated by significantly upregulated renal gene expression of *TNFA* and *IL10* in the preterm control group, relative to the term group (*p* < 0.05 and *p* < 0.001, respectively). Treatment with IGF-1 resulted in significant decreases in renal *TNFA* (*p* < 0.05) and *IL10* (*p* < 0.001) expression in preterm pigs on PND5 (Fig. 5a, b). Renal protein levels of TNFα and IL10 exhibited a trend similar to that of their respective mRNA levels on PND5, although the differences did not reach significance (Fig. 5d, e). The *IL6* gene expression did not differ significantly between the groups (*p* > 0.05, Fig. 5c).

Pearson correlation analysis revealed a strong positive correlation between the gene expression levels related to kidney inflammation and those related to kidney injury on PND5 (Supplemental Figure S5), suggesting that preterm birth-associated kidney injury is closely related to increased local inflammatory responses.

## Discussion

Our findings demonstrate the effects of preterm birth on nephrogenesis, with a heightened risk of abnormal glomerular development in the postnatal period in preterm pigs. By comparing cesarean-delivered, artificially reared preterm pigs with vaginally born, sow-reared term pigs, we described the combined effects of reduced gestational age at birth, delivery method and postnatal environment on kidney development. In humans, nephrogenesis is believed to start at 5 weeks gestation and continue until around 36 weeks, with the most rapid development occurring during the third trimester.^41,55,56^ However, nephrogenesis can still occur from week 36 to 40 in humans.^43^ In pigs, nephrogenesis begins at 4 weeks gestation and ends around 3 weeks after birth.^57,58^ Based on this time course, preterm pigs delivered at 90% of gestational age (approximately 70% completion of nephrogenesis) may have a kidney maturation state similar to that of infants with a gestational age of around 27-30 weeks. However, a more accurate assessment of kidney maturation stages in pigs compared to humans requires further investigation as there is no detailed information regarding the velocity of swine nephrogenesis at different developmental stages, and pigs may adapt to postnatal kidney maturation in a species-specific manner. Although the study did not adequately determine renal function, it indicates that preterm birth has clear adverse effects on kidney structural development. Notably, the deficiencies in kidney structure and histopathology observed at the first week after preterm birth could be partially ameliorated by IGF-1 supplementation. Thus, IGF-1 could serve as a promising therapeutic target for kidney immaturity in preterm infants.

Preterm birth is associated with reduced nephron endowment.^59^ Compared to term pigs, a lower nephron number in preterm control pigs, as reflected by a lower glomerular generation number^60^ and density, might be due to missing critical steps of normal nephrogenesis in the uterus. The smaller glomerular area in preterm control animals might also be due to insufficient glomerular development or perfusion, as previously shown in preterm lambs.^54^ Preterm birth might initiate a compensatory mechanism of nephron formation, as indicated by increased NZW and the expression of genes related to nephrogenesis, including *GDNF* (a ureteric bud branching marker), *WNT9B* (a key molecule in nephron morphogenesis^61^) and *SIX2* (a nephron progenitor marker^62,63^). This adaptation might enable the kidney to quickly adjust to the increased demands of the extrauterine environment.^1,41^ Notably, a significant proportion of morphologically abnormal glomeruli were observed in preterm pigs particularly on PND5. These abnormal glomeruli may temporarily form in response to the stresses of the extrauterine environment,^49^ as evidenced by the observation that their proportion decreases on PND9 and 19.

Preterm newborns are susceptible to AKI due to their immature renal structure and function.^7,9,64,65^ AKI typically occurs within the first week of postnatal life.^66–68^ These findings align with our observation that the kidney of preterm pigs appear to be most susceptible to inflammation and injury during the first week after birth. Kidney injury is closely related to intrarenal and systemic inflammation.^69–71^ The histological findings of our study resemble those seen in a cecal ligation and puncture-induced septic rat model.^72^ Since severe NEC with systemic inflammation and hemodynamic instability predisposes preterm infants to AKI,^22,73–75^ we further investigated whether relatively mild NEC can also affect premature kidneys. However, our findings indicate that the kidney injury was primarily associated with intrarenal inflammation instead of mild NEC.

In preterm infants, lower levels of circulating IGF-1 are associated with fetal and postnatal growth restriction, systemic inflammation and complications related to multiple organs.^33^ A large multicenter international trial is currently underway to determine whether the administration of supplemental IGF-1 can reduce morbidities in extremely preterm infants (ClinicalTrials.gov registry NCT03253263), partly based on the initial evidence of reduced bronchopulmonary dysplasia and intraventricular hemorrhage.^76^ In the present study, we demonstrate that supplemental IGF-1 treatment may promote nephrogenesis and alleviate preterm birth-associated renal impairments. Correlation analyses between kidney variables and NEC lesion scores suggest that IGF-1 treatment exerts direct effects on the immature kidney without interacting with gut-related inflammation. The promotive effects of IGF-1 on immature kidney were mainly supported by the morphological findings, together with the upregulated expression of key nephrogenesis-related genes, including *GDNF*, *CTNNB1*, *WNT9B*, *TGFB1* (involved in elongation of the ureteric bud and glomerular capillary formation^77–79^) and *AT1* (associated with normal tubular maturation^80^) on PND5. IGF-1 plays a crucial role in the development of many organ systems, likely through its effects on vascular development.^25,30,81–85^ Microvascular growth is essential for nephron formation. The abnormal glomeruli observed in preterm infants are believed to result from compromised capillarization due to accelerated adaptation to the extrauterine environment.^49,54^ Our findings of a decreased percentage of abnormal glomeruli and a larger glomerular area after IGF-1 treatment suggest that IGF-1 may promote glomerular microvascular formation in the immature kidney. Additionally, IGF-1 exhibits antiapoptotic and mitogenic characteristics,^86–89^ which may explain the protective effect of IGF-1 against potential kidney injury in preterm pigs. Both the activation of IGF-1 and the upregulation of *TGFB1* are known to promote kidney fibrosis in various disease conditions.^90,91^ However, since we found no increase in FMA in IGF-1 treated kidneys, we conclude that IGF-1 may not increase the risk of fibrosis by upregulating *TGFB1* expression in preterm pigs. Nevertheless, further investigation is needed to ensure the safety of IGF-1 administration in neonates.

There are limitations to our study. It is important to note that our preterm-term comparisons relate not only to the effects of reduced gestational age at birth but also to the differences in the external rearing environment, similar to any clinical trial comparing hospitalized preterm infants and mother-reared term infants. Furthermore, the biochemical data presented in the study does not offer comprehensive insights into alterations in kidney function. While serum creatinine has traditionally been used as the main parameter to determine renal function in newborns,^92^ it is recognized that a single-point serum creatinine measurement is not sufficient for diagnosing kidney dysfunction in newborns due to the significant influence of maternal creatinine levels on that in neonates.^93,94^ However, since we adjusted the litter (mother) factor via the linear model when comparing the preterm control and the preterm IGF-1 groups, we believe that the plasma creatinine levels can be effectively used to relatively reflect the kidney function in preterm pigs. Besides, we calculated the eGFR in piglets based on a formula established in pigs using plasma creatinine levels.^39^ We further use BUN levels as an indicator of immature renal function during early life.^95^ As our results revealed no significant differences in plasma creatinine, eGFR and BUN between the preterm control and the preterm IGF-1 groups, it can be cautiously inferred that the administration of IGF-1 does not induce fundamental alterations in kidney function among preterm pigs. However, it is unclear whether there were more subtle changes of kidney function or changes during later stages of life, which were beyond the scope of this study. Additionally, comparing kidney function between term and preterm pigs using plasma creatinine, eGFR and BUN levels presents challenging, because term pigs were delivered by different sows and under natural suckling conditions. As neonatal plasma creatinine is influenced not only by maternal creatinine levels^93,94^ but also by muscle mass and activity,^96^ while BUN levels can be primarily affected by protein intake and amino acid metabolic levels.^97^ To comprehensively address these limitations, further in-depth studies are required, with a specific focus on evaluating kidney function before and after IGF-1 supplementation. This could be achieved through a longer-term animal study with continuous recording of GFR, plasma creatinine (maternal/neonatal), and urine output.^93,94^ Despite these limitations, our study has provided significant new preclinical evidence regarding the effects of supplemental IGF-1 on kidney development following preterm birth. This animal model can currently be used to investigate short- and long-term kidney effects following various adverse conditions related to preterm birth, including experimental birth asphyxia, growth-restriction before and after birth,^98,99^ perinatal inflammation^100^ and neonatal sepsis.^101^

## Supplementary information

Supplementary Information

## Data Availability

All datasets generated during and analyzed during the current study, including raw data used for all figures, tables and analysis, are available from the corresponding author on reasonable request.

## References

[1] Black, M. J. et al. When Birth Comes Early: Effects on Nephrogenesis. *Nephrology* **18**, 180–182 (2013). 23279726 10.1111/nep.12028

[2] Blencowe, H. et al. National, Regional, and Worldwide Estimates of Preterm Birth Rates in the Year 2010 with Time Trends since 1990 for Selected Countries: A Systematic Analysis and Implications. *lancet* **379**, 2162–2172 (2012). 22682464 10.1016/S0140-6736(12)60820-4

[3] Zierden, H. C., Shapiro, R. L., DeLong, K., Carter, D. M. & Ensign, L. M. Next Generation Strategies for Preventing Preterm Birth. *Adv. Drug Deliv. Rev.* **174**, 190–209 (2021). 33895215 10.1016/j.addr.2021.04.021 PMC8217279

[4] Gubhaju, L. et al. Is Nephrogenesis Affected by Preterm Birth? Studies in a Non-Human Primate Model. *Am. J. Physiol. Ren. Physiol.* **297**, F1668–F1677 (2009). 10.1152/ajprenal.00163.2009 PMC2801336 19759270

[5] Sutherland, M. R. et al. Renal Dysfunction Is Already Evident within the First Month of Life in Australian Indigenous Infants Born Preterm. *Kidney Int* **96**, 1205–1216 (2019). 31563332 10.1016/j.kint.2019.07.015

[6] Awad, H., El-Barbary, M., Imam, S. & El-Safty, I. Evaluation of Renal Glomerular and Tubular Functional and Structural Integrity in Neonates. *Am. J. Med. Sci.* **324**, 261–266 (2002). 12449447 10.1097/00000441-200211000-00005

[7] Sulemanji, M. & Vakili, K. in *Semin. Pediatr. Surg*. 195-198 (Elsevier). 10.1053/j.sempedsurg.2013.10.008 24331094

[8] Gubhaju, L. et al. Assessment of Renal Functional Maturation and Injury in Preterm Neonates During the First Month of Life. *Am. J. Physiol. Renal Physiol*. (2014). 10.1152/ajprenal.00439.2013 24899060

[9] Luyckx, V. A. in *Semin. Nephrol*. 311-319 (Elsevier). 10.1016/j.semnephrol.2017.05.002 28711069

[10] Charlton, J. R. et al. Late Onset Neonatal Acute Kidney Injury: Results from the Awaken Study. *Pediatr. Res.* **85**, 339–348 (2019). 30546043 10.1038/s41390-018-0255-x PMC6438709

[11] Stritzke, A., Thomas, S., Amin, H., Fusch, C. & Lodha, A. Renal Consequences of Preterm Birth. *Mol. Cell. Pediatr.* **4**, 1–9 (2017). 28101838 10.1186/s40348-016-0068-0 PMC5243236

[12] Kuure, S., Vuolteenaho, R. & Vainio, S. Kidney Morphogenesis: Cellular and Molecular Regulation. *Mech. Dev.* **92**, 31–45 (2000). 10704886 10.1016/s0925-4773(99)00323-8

[13] Cullen-McEwen, L., Sutherland, M. R. & Black, M. J. in *Kidney Development, Disease, Repair and Regeneration* 27-40 (Elsevier, 2016).

[14] Faa, G. et al. Morphogenesis and Molecular Mechanisms Involved in Human Kidney Development. *J. Cell. Physiol.* **227**, 1257–1268 (2012). 21830217 10.1002/jcp.22985

[15] Costantini, F. & Shakya, R. Gdnf/Ret Signaling and the Development of the Kidney. *Bioessays* **28**, 117–127 (2006). 16435290 10.1002/bies.20357

[16] Wang, Y., Zhou, C. J. & Liu, Y. Wnt Signaling in Kidney Development and Disease. *Prog. Mol. Biol. Transl. Sci.* **153**, 181–207 (2018). 29389516 10.1016/bs.pmbts.2017.11.019 PMC6008255

[17] Humberg, A. et al. in *Semin. Immunopathol*. 451-468 (Springer). 10.1007/s00281-020-00803-2 PMC7508934 32661735

[18] Galinsky, R. et al. Effect of Intra-Amniotic Lipopolysaccharide on Nephron Number in Preterm Fetal Sheep. *Am. J. Physiol. Ren. Physiol.* **301**, F280–F285 (2011). 10.1152/ajprenal.00066.2011 21593183

[19] Neu, J. & Walker, W. A. Necrotizing Enterocolitis. *N. Engl. J. Med.* **364**, 255–264 (2011). 21247316 10.1056/NEJMra1005408 PMC3628622

[20] Thompson, A. M. & Bizzarro, M. J. Necrotizing Enterocolitis in Newborns. *Drugs* **68**, 1227–1238 (2008). 18547133 10.2165/00003495-200868090-00004

[21] Srinivasan, P. S., Brandler, M. D. & D’Souza, A. Necrotizing Enterocolitis. *Clin. Perinatol.* **35**, 251–272 (2008). 18280885 10.1016/j.clp.2007.11.009

[22] Bakhoum, C. Y., Basalely, A., Koppel, R. I. & Sethna, C. B. Acute Kidney Injury in Preterm Infants with Necrotizing Enterocolitis. *J. Matern. Fetal Neonatal Med.* **32**, 3185–3190 (2019). 29631454 10.1080/14767058.2018.1459553

[23] Edelson, M. B., Bagwell, C. E. & Rozycki, H. J. Circulating Pro-and Counterinflammatory Cytokine Levels and Severity in Necrotizing Enterocolitis. *Pediatrics* **103**, 766–771 (1999). 10103300 10.1542/peds.103.4.766

[24] Laviola, L., Natalicchio, A., Perrini, S. & Giorgino, F. Abnormalities of Igf-I Signaling in the Pathogenesis of Diseases of the Bone, Brain, and Fetoplacental Unit in Humans. *Am. J. Physiol. - Endocrinol. Metab.* **295**, E991–E999 (2008). 18713961 10.1152/ajpendo.90452.2008

[25] Hellström, A. et al. Role of Insulinlike Growth Factor 1 in Fetal Development and in the Early Postnatal Life of Premature Infants. *Am. J. Perinatol.* **33**, 1067–1071 (2016). 27603537 10.1055/s-0036-1586109 PMC5779855

[26] Rajaram, S., Baylink, D. J. & Mohan, S. Insulin-Like Growth Factor-Binding Proteins in Serum and Other Biological Fluids: Regulation and Functions. *Endocr. Rev.* **18**, 801–831 (1997). 9408744 10.1210/edrv.18.6.0321

[27] Hellstrom, A. et al. Igf-1 as a Drug for Preterm Infants: A Step-Wise Clinical Development. *Curr. Pharm. Des.* **23**, 5964–5970 (2017). 28969546 10.2174/1381612823666171002114545 PMC5824464

[28] Bach, L. A. & Hale, L. J. Insulin-Like Growth Factors and Kidney Disease. *Am. J. Kidney Dis.* **65**, 327–336 (2015). 25151409 10.1053/j.ajkd.2014.05.024

[29] Cingel-Ristić, V., Flyvbjerg, A. & Drop, S. L. The Physiological and Pathophysiological Roles of the Gh/Igf-Axis in the Kidney: Lessons from Experimental Rodent Models. *Growth Horm. IGF Res.* **14**, 418–430 (2004). 15519249 10.1016/j.ghir.2004.06.003

[30] Lumbers, E. R. et al. Effects of Intrafetal Igf-I on Growth of Cardiac Myocytes in Late-Gestation Fetal Sheep. *Am. J. Physiol. - Endocrinol. Metab.* **296**, E513–E519 (2009). 19126787 10.1152/ajpendo.90497.2008

[31] Fernández, M. et al. Exacerbated Inflammatory Response Induced by Insulin-Like Growth Factor I Treatment in Rats with Ischemic Acute Renal Failure. *J. Am. Soc. Nephrol.* **12**, 1900–1907 (2001). 11518783 10.1681/ASN.V1291900

[32] Holgersen, K. et al. Supplemental Insulin-Like Growth Factor-1 and Necrotizing Enterocolitis in Preterm Pigs. *Front. Pediatr.* **8**, 602047 (2021). 33614541 10.3389/fped.2020.602047 PMC7891102

[33] Holgersen, K. et al. Clinical Outcome and Gut Development after Insulin-Like Growth Factor-1 Supplementation to Preterm Pigs. *Front. Pediatr*. **10**, 868911 (2022). 10.3389/fped.2022.868911 PMC9389362 35989990

[34] Rasmussen, et al. Gut Development Following Insulin-Like Growth Factor-1 Supplementation to Preterm Pigs. *Pediatr. Res*. 10.1038/s41390-023-02949-9 (2023). 10.1038/s41390-023-02949-9 PMC11126387 38086951

[35] Tavakkol, A., Simmen, F. A. & Simmen, R. C. Porcine Insulin-Like Growth Factor-I (Pigf-I): Complementary Deoxyribonucleic Acid Cloning and Uterine Expression of Messenger Ribonucleic Acid Encoding Evolutionary Conserved Igf-I Peptides. *Mol. Endocrinol.* **2**, 674–681 (1988). 3211153 10.1210/mend-2-8-674

[36] Cilieborg, M. S., Boye, M., Thymann, T., Jensen, B. B. & Sangild, P. T. Diet‐Dependent Effects of Minimal Enteral Nutrition on Intestinal Function and Necrotizing Enterocolitis in Preterm Pigs. *J. Parenter. Enter. Nutr.* **35**, 32–42 (2011). 10.1177/0148607110377206 21224432

[37] Sangild, P. T. et al. Invited Review: The Preterm Pig as a Model in Pediatric Gastroenterology. *J. Anim. Sci.* **91**, 4713–4729 (2013). 23942716 10.2527/jas.2013-6359 PMC3984402

[38] Yan, X. et al. Supplementary Bovine Colostrum Feedings to Formula-Fed Preterm Pigs Improve Gut Function and Reduce Necrotizing Enterocolitis. *J. Pediatr. Gastroenterol. Nutr.* **73**, e39–e46 (2021). 33853107 10.1097/MPG.0000000000003147

[39] Gasthuys, E. et al. Postnatal Maturation of the Glomerular Filtration Rate in Conventional Growing Piglets as Potential Juvenile Animal Model for Preclinical Pharmaceutical Research. *Front. Pharmacol.* **8**, 431 (2017). 28706488 10.3389/fphar.2017.00431 PMC5489626

[40] Popescu, C. R. et al. Hyperoxia Exposure Impairs Nephrogenesis in the Neonatal Rat: Role of Hif-1α. *PLoS One* **8**, e82421 (2013). 24358181 10.1371/journal.pone.0082421 PMC3866112

[41] Sutherland, M. R. et al. Accelerated Maturation and Abnormal Morphology in the Preterm Neonatal Kidney. *J. Am. Soc. Nephrol.* **22**, 1365–1374 (2011). 21636639 10.1681/ASN.2010121266 PMC3137584

[42] Sutherland, M. R. et al. Effects of Ibuprofen Treatment on the Developing Preterm Baboon Kidney. *Am. J. Physiol. Ren. Physiol.* **302**, F1286–F1292 (2012). 10.1152/ajprenal.00216.2011 PMC3362063 22357916

[43] dos Santos, A. M. et al. Assessment of Renal Maturity by Assisted Morphometry in Autopsied Fetuses. *Early Hum. Dev.* **82**, 709–713 (2006). 16687220 10.1016/j.earlhumdev.2006.01.013

[44] Rodríguez, M. M. et al. Histomorphometric Analysis of Postnatal Glomerulogenesis in Extremely Preterm Infants. *Pediatr. Dev. Pathol.* **7**, 17–25 (2004). 15255031 10.1007/s10024-003-3029-2

[45] Hinchliffe, S. et al. “Medullary Ray Glomerular Counting” as a Method of Assessment of Human Nephrogenesis. *Pathol. Res. Pract.* **188**, 775–782 (1992). 1437841 10.1016/S0344-0338(11)80177-9

[46] Bassan, H. et al. Experimental Intrauterine Growth Retardation Alters Renal Development. *Pediatr. Nephrol.* **15**, 192–195 (2000). 11149109 10.1007/s004670000457

[47] Abitbol, C. L., DeFreitas, M. J. & Strauss, J. Assessment of Kidney Function in Preterm Infants: Lifelong Implications. *Pediatr. Nephrol.* **31**, 2213–2222 (2016). 26846786 10.1007/s00467-016-3320-x

[48] Koike, K. et al. Glomerular Density and Volume in Renal Biopsy Specimens of Children with Proteinuria Relative to Preterm Birth and Gestational Age. *Clin. J. Am. Soc. Nephrol.* **12**, 585–590 (2017). 28336816 10.2215/CJN.05650516 PMC5383381

[49] Sutherland, M. R., Gubhaju, L., Yoder, B. A., Stahlman, M. T. & Black, M. J. The Effects of Postnatal Retinoic Acid Administration on Nephron Endowment in the Preterm Baboon Kidney. *Pediatr. Res.* **65**, 397–402 (2009). 19092718 10.1203/PDR.0b013e3181975f52 PMC3633555

[50] Tanaka, T. & Nangaku, M. Angiogenesis and Hypoxia in the Kidney. *Nat. Rev. Nephrol.* **9**, 211–222 (2013). 23458926 10.1038/nrneph.2013.35

[51] Bock, F. et al. Activated Protein C Ameliorates Diabetic Nephropathy by Epigenetically Inhibiting the Redox Enzyme P66shc. *Proc. Natl Acad. Sci. Usa.* **110**, 648–653 (2013). 23267072 10.1073/pnas.1218667110 PMC3545757

[52] Sun, J. et al. Ultra‐High Temperature Treatment of Liquid Infant Formula, Systemic Immunity, and Kidney Development in Preterm Neonates. *Mol. Nutr. Food Res.* **67**, 2300318 (2023). 10.1002/mnfr.202300318 37888862

[53] Wang, S., Wang, B., He, H., Sun, A. & Guo, C. A New Set of Reference Housekeeping Genes for the Normalization Rt-Qpcr Data from the Intestine of Piglets During Weaning. *PLoS One* **13**, e0204583 (2018). 30256841 10.1371/journal.pone.0204583 PMC6157878

[54] Sutherland, M. R., Danica, R., Dahl, M. J., Albertine, K. H. & Black, M. J. Effects of Preterm Birth and Ventilation on Glomerular Capillary Growth in the Neonatal Lamb Kidney. *J. Hypertens.* **34**, 1988 (2016). 27428042 10.1097/HJH.0000000000001028 PMC5683853

[55] Mackenzie, H. S. & Brenner, B. M. Fewer Nephrons at Birth: A Missing Link in the Etiology of Essential Hypertension? *Am. J. Kidney Dis.* **26**, 91–98 (1995). 7611275 10.1016/0272-6386(95)90161-2

[56] Hinchliffe, S., Sargent, P., Howard, C., Chan, Y. & Van Velzen, D. Human Intrauterine Renal Growth Expressed in Absolute Number of Glomeruli Assessed by the Disector Method and Cavalieri Principle. *Lab. Invest.* **64**, 777–784 (1991). 2046329

[57] Friis, C. Postnatal Development of the Pig Kidney: Ultrastucure of the Glomerulus and the Proximal Tubule. *J. Anat.* **130**, 513 (1980). 7410196 PMC1233171

[58] Egerer, G., Taugner, R. & Tiedemann, K. Renin Immunohistochemistry in the Mesonephros and Metanephros of the Pig Embryo. *Histochemistry* **81**, 385–390 (1984). 6392218 10.1007/BF00514334

[59] Black, M. J., Sutherland, M. R. & Gubhaju, L. in *Basic Nephrology and Acute Kidney Injury*. 61–88 (2012).

[60] Sutherland, M. R., Gubhaju, L. & Black, M. J. Stereological Assessment of Renal Development in a Baboon Model of Preterm Birth. *Am. J. Nephrol.* **33**, 25–33 (2011). 21659732 10.1159/000327073

[61] Karner, C. M. et al. Wnt9b Signaling Regulates Planar Cell Polarity and Kidney Tubule Morphogenesis. *Nat. Genet.* **41**, 793–799 (2009). 19543268 10.1038/ng.400 PMC2761080

[62] Faraj, R., Irizarry-Alfonzo, A. & Puri, P. Molecular Characterization of Nephron Progenitors and Their Early Epithelial Derivative Structures in the Nephrogenic Zone of the Canine Fetal Kidney. *Eur. J. Histochem*. **63**, 158–168 (2019). 10.4081/ejh.2019.3049 PMC6763752 31544449

[63] Hendry, C., Rumballe, B., Moritz, K. & Little, M. H. Defining and Redefining the Nephron Progenitor Population. *Pediatr. Nephrol.* **26**, 1395–1406 (2011). 21229268 10.1007/s00467-010-1750-4 PMC3189495

[64] Stojanović, V., Barišić, N., Milanović, B. & Doronjski, A. Acute Kidney Injury in Preterm Infants Admitted to a Neonatal Intensive Care Unit. *Pediatr. Nephrol.* **29**, 2213–2220 (2014). 24839217 10.1007/s00467-014-2837-0

[65] Hanna, M. et al. Early Urinary Biomarkers of Acute Kidney Injury in Preterm Infants. *Pediatr. Res.* **80**, 218–223 (2016). 27055185 10.1038/pr.2016.70

[66] Weintraub, A., Connors, J., Carey, A., Blanco, V. & Green, R. The Spectrum of Onset of Acute Kidney Injury in Premature Infants Less Than 30 Weeks Gestation. *J. Perinatol.* **36**, 474–480 (2016). 26796125 10.1038/jp.2015.217

[67] Charlton, J. R. et al. Incidence and Risk Factors of Early Onset Neonatal Aki. *Clin. J. Am. Soc. Nephrol.* **14**, 184–195 (2019). 31738181 10.2215/CJN.03670318 PMC6390916

[68] Starr, M. C. et al. Advances in Neonatal Acute Kidney Injury. *Pediatrics* **148**, e2021051220 (2021). 10.1542/peds.2021-051220 34599008

[69] Rabb, H. et al. Inflammation in Aki: Current Understanding, Key Questions, and Knowledge Gaps. *J. Am. Soc. Nephrol.* **27**, 371–379 (2016). 26561643 10.1681/ASN.2015030261 PMC4731128

[70] Kinsey, G. R., Li, L. & Okusa, M. D. Inflammation in Acute Kidney Injury. *Nephron Exp. Nephrol.* **109**, e102–e107 (2008). 18802372 10.1159/000142934 PMC2614446

[71] Hoogenboom, L. A. et al. Chorioamnionitis Causes Kidney Inflammation, Podocyte Damage, and Pro-Fibrotic Changes in Fetal Lambs. *Front. Pediatr.* **10**, 390 (2022). 10.3389/fped.2022.796702 PMC9013807 35444963

[72] Lin, Z., Jin, J. & Shan, X. Fish Oils Protects against Cecal Ligation and Puncture‑Induced Septic Acute Kidney Injury Via the Regulation of Inflammation, Oxidative Stress and Apoptosis. *Int. J. Mol. Med.* **44**, 1771–1780 (2019). 31545434 10.3892/ijmm.2019.4337 PMC6777667

[73] Sánchez, C., García, M. A. & Valdés, B. D. Acute Kidney Injury in Newborns with Necrotizing Enterocolitis: Risk Factors and Mortality. *Bol. Med. Hosp. Infant. Mex.* **76**, 210–214 (2019). 31536045 10.24875/BMHIM.19000044

[74] Garg, P. M. et al. Severe Acute Kidney Injury in Neonates with Necrotizing Enterocolitis: Risk Factors and Outcomes. *Pediatr. Res.* **90**, 642–649 (2021). 33446918 10.1038/s41390-020-01320-6 PMC8277891

[75] Garg, P. M. et al. Clinical Impact of Nec-Associated Sepsis on Outcomes in Preterm Infants. *Pediatr. Res.* **92**, 1705–1715 (2022). 35352003 10.1038/s41390-022-02034-7 PMC10311923

[76] Ley, D. et al. Rhigf-1/Rhigfbp-3 in Preterm Infants: A Phase 2 Randomized Controlled Trial. *J. Pediatr.* **206**, 56–65.e58 (2019). 30471715 10.1016/j.jpeds.2018.10.033 PMC6389415

[77] Akison, L. K. et al. Moderate Prenatal Ethanol Exposure in the Rat Promotes Kidney Cell Apoptosis, Nephron Deficits, and Sex‐Specific Kidney Dysfunction in Adult Offspring. *Anat. Rec.* **303**, 2632–2645 (2020). 10.1002/ar.24370 31984647

[78] Ritvos, O. et al. Activin Disrupts Epithelial Branching Morphogenesis in Developing Glandular Organs of the Mouse. *Mech. Dev.* **50**, 229–245 (1995). 7619733 10.1016/0925-4773(94)00342-k

[79] Liu, A., Dardik, A. & Ballermann, B. J. Neutralizing Tgf-Β1 Antibody Infusion in Neonatal Rat Delays in Vivo Glomerular Capillary Formation. *Kidney Int* **56**, 1334–1348 (1999). 10504486 10.1046/j.1523-1755.1999.00661.x

[80] Chen, Y. et al. Neonatal Losartan Treatment Suppresses Renal Expression of Molecules Involved in Cell-Cell and Cell-Matrix Interactions. *J. Am. Soc. Nephrol.* **15**, 1232–1243 (2004). 15100363 10.1097/01.asn.0000123690.75029.3f

[81] Moerth, C. et al. Postnatally Elevated Levels of Insulin-Like Growth Factor (Igf)-Ii Fail to Rescue the Dwarfism of Igf-I-Deficient Mice except Kidney Weight. *Endocrinology* **148**, 441–451 (2007). 17008389 10.1210/en.2006-0385

[82] Yan, X., Managlia, E., Zhao, Y.-Y., Tan, X.-D. & De Plaen, I. G. Macrophage-Derived Igf-1 Protects the Neonatal Intestine against Necrotizing Enterocolitis by Promoting Microvascular Development. *Commun. Biol.* **5**, 320 (2022). 35388142 10.1038/s42003-022-03252-9 PMC8987083

[83] Kramer, B. W., Niklas, V. & Abman, S. Bronchopulmonary Dysplasia and Impaired Neurodevelopment—What May Be the Missing Link? *Am. J. Perinatol.* **39**, S14–S17 (2022). 36318942 10.1055/s-0042-1756677

[84] Wang, Z. et al. Insulin-Like Growth Factor-1 Signaling in Lung Development and Inflammatory Lung Diseases. *Biomed Res. Int*. **2018**, 6057589 (2018). 10.1155/2018/6057589 PMC6029485 30018981

[85] Smith, L. E. Igf-1 and Retinopathy of Prematurity in the Preterm Infant. *Neonatology* **88**, 237–244 (2005). 10.1159/000087587 16210846

[86] Miller, S. B., Martin, D. R., Kissane, J. & Hammerman, M. R. Insulin-Like Growth Factor I Accelerates Recovery from Ischemic Acute Tubular Necrosis in the Rat. *Proc. Natl Acad. Sci. Usa.* **89**, 11876–11880 (1992). 1465411 10.1073/pnas.89.24.11876 PMC50660

[87] Hirschberg, R. & Adler, S. Insulin-Like Growth Factor System and the Kidney: Physiology, Pathophysiology, and Therapeutic Implications. *Am. J. Kidney Dis.* **31**, 901–919 (1998). 9631833 10.1053/ajkd.1998.v31.pm9631833

[88] Zang, H., Yang, Q. & Li, J. Eleutheroside B Protects against Acute Kidney Injury by Activating Igf Pathway. *Molecules* **24**, 3876 (2019). 31661774 10.3390/molecules24213876 PMC6864713

[89] Imberti, B. et al. Insulin-Like Growth Factor-1 Sustains Stem Cell–Mediated Renal Repair. *J. Am. Soc. Nephrol.* **18**, 2921–2928 (2007). 17942965 10.1681/ASN.2006121318

[90] Tampe, B. & Zeisberg, M. Contribution of Genetics and Epigenetics to Progression of Kidney Fibrosis. *Nephrol. Dial. Transplant.* **29**, iv72–iv79 (2014). 23975750 10.1093/ndt/gft025

[91] Dong, R. et al. Igf-1/Igf-1r Blockade Ameliorates Diabetic Kidney Disease through Normalizing Snail1 Expression in a Mouse Model. *Am. J. Physiol. Endocrinol. Metab.* **317**, E686–E698 (2019). 31361542 10.1152/ajpendo.00071.2019

[92] Sandinirwan, I., Primadi, A. & Hilmanto, D. Serum Creatinine Levels to Estimate Kidney Function in Small-for-Gestational Age and Appropriate-for-Gestational Age Newborns. *Paediatr. Indones.* **58**, 305–311 (2018).

[93] Nada, A., Bonachea, E. M. & Askenazi, D. J. in *Semin. Fetal Neonatal Med*. 90-97 (Elsevier). 10.1016/j.siny.2016.12.001 PMC5373985 28034548

[94] Chen, J. et al. The Effectiveness of Urinary Timp-2 and Igfbp-7 in Predicting Acute Kidney Injury in Critically Ill Neonates. *Pediatr. Res.* **87**, 1052–1059 (2020). 31791043 10.1038/s41390-019-0698-8

[95] Weintraub, A., Blanco, V., Barnes, M. & Green, R. Impact of Renal Function and Protein Intake on Blood Urea Nitrogen in Preterm Infants in the First 3 Weeks of Life. *J. Perinatol.* **35**, 52–56 (2015). 25078864 10.1038/jp.2014.138

[96] Cala, A. et al. Influence of Muscle Mass and Physical Activity on Serum and Urinary Creatinine and Serum Cystatin C. *Clin. J. Am. Soc. Nephrol.* **3**, 348–354 (2008). 18235143 10.2215/CJN.02870707 PMC2390952

[97] Roggero, P. et al. Blood Urea Nitrogen Concentrations in Low-Birth-Weight Preterm Infants During Parenteral and Enteral Nutrition. *J. Pediatr. Gastroenterol. Nutr.* **51**, 213–215 (2010). 20479690 10.1097/MPG.0b013e3181cd270f

[98] Bæk, O., Ren, S., Brunse, A., Sangild, P. T. & Nguyen, D. N. Impaired Neonatal Immunity and Infection Resistance Following Fetal Growth Restriction in Preterm Pigs. *Front. Immunol.* **11**, 1808 (2020). 32903565 10.3389/fimmu.2020.01808 PMC7438575

[99] Bæk, O., Sangild, P. T., Thymann, T. & Nguyen, D. N. Growth Restriction and Systemic Immune Development in Preterm Piglets. *Front. Immunol.* **10**, 2402 (2019). 31649685 10.3389/fimmu.2019.02402 PMC6795705

[100] Muk, T. et al. Prenatal Endotoxin Exposure Induces Fetal and Neonatal Renal Inflammation Via Innate and Th1 Immune Activation in Preterm Pigs. *Front. Immunol.* **11**, 565484 (2020). 33193334 10.3389/fimmu.2020.565484 PMC7643587

[101] Muk, T., Brunse, A., Henriksen, N. L., Aasmul-Olsen, K. & Nguyen, D. N. Glucose Supply and Glycolysis Inhibition Shape the Clinical Fate of Staphylococcus Epidermidis-Infected Preterm Newborns. *JCI Insight* **7**, e157234 (2022). 10.1172/jci.insight.157234 PMC9220920 35503431

