# Supplementary material for: Insulin-like growth factor-1 effects on kidney development in preterm piglets
Source: Pediatr Res. 2024 May 18;96(7):1655–65. doi: 10.1038/s41390-024-03222-3 (PMC11772250; doi:10.1038/s41390-024-03222-3)

Supplemental files

Insulin-Like Growth Factor-1 Effects on Kidney Development in Preterm Piglets

Jingren Zhong[1], Richard Doughty[2], Thomas Thymann[1], Per Torp Sangild[1,3,4], Duc Ninh Nguyen[1] and Tik Muk[1*]

Affiliations:

1. Section for Comparative Paediatrics and Nutrition, Department of Veterinary and Animal Sciences, University of Copenhagen, Frederiksberg, Denmark
2. Department of Pathology, Akershus University Hospital, Lørenskog, Norway
3. Department of Pediatrics, Odense University Hospital, Odense, Denmark
4. Department of Neonatology, Rigshospitalet, Copenhagen, Denmark

***Correspondence:** Dr. Tik Muk, Section for Comparative Pediatrics and Nutrition, Department of Veterinary and Animal Sciences, Faculty of Health and Medical Sciences, University of Copenhagen, Dyrlægevej 68, DK-1870 Frederiksberg C, Denmark, Tel: + 4535332272, email: tik.muk@sund.ku.dk

Category of study: Basic science

Impact:

1. Preterm birth may disrupt kidney development in newborns, potentially leading to morphological changes, injury, and inflammation.
2. Preterm pigs have previously been used as models for preterm infants, but not for kidney development.
3. IGF-1 supplementation promotes kidney maturation and alleviates renal impairments in the first week of life in preterm pigs. IGF-1 may hold potential as a supportive therapy for preterm infants sensitive to acute kidney injury.

Supplemental Table S1 List of gene names, primer sequences and amplicon length of primers used in the study.

Gene name	Gene symbol	Sequence (5' to 3')	Sequence (3' to 5')	Amplicon length
HPRT1	Hypoxanthine phosphoribosyltransferase 1	TATGGACAGGACTGAACGGC	ATCCAGCAGGTCAGCAAAGA	115
CASPASE3	Caspase 3	CCGGAATGGCATGTCGATCT	CATGGCTTAGAAGCACGCAA	189
GATA3	GATA binding protein 3	ACCCCTTATTAAGCCCAAGC	TCCAGAGAGTCGTCGTTGTG	92
IL10	Interleukin 10	GTCCGACTCAACGAAGAAGG	GCCAGGAAGATCAGGCAATA	73
IL6	Interleukin 6	TGGGTTCAATCAGGAGACCT	CAGCCTCGACATTTCCCTTA	116
TBET	T-box transcription factor 21	CTGAGAGTCGCGCTCAACAA	ACCCGGCCACAGTAAATGAC	121
TNFA	Tumor necrosis factor alpha	ATTCAGGGATGTGTGGCCTG	CCAGATGTCCCAGGTTGCAT	120
HIF1A	Hypoxia-inducible factor 1 alpha	TGTGTTATCTGTCGCTTTGAGTC	TTTCGCTTTCTCTGAGCATTC	96
KIM1	Kidney injury molecule-1	ATGTACCCTTGGGTAACCGC	AACGTAGAACATGCCCCTCG	164
NGAL	Neutrophil gelatinase-associated lipocalin	AAGACGGCAGCTACAACGTC	GACACCACACGCACGACATA	157
LRG1	Leucine Rich Alpha-2-Glycoprotein 1	TGACCTGCACATCCTTGACC	CAGAAAGCCCTCTTCGAGCA	133
IGFBP7	Insulin like growth factor binding protein 7	ATCGTGACACCCCCTAAGGAC	ATAGTGACCCCTTTGTACCTTGT	120
WNT11	Wnt family member 11	CTTGACCTGGAGAGAGGGACC	ATGAGGAGCCCGTAGCTGAG	197
WNT4	Wnt family member 4	CACAAGGCTTCCAGTGGTCA	GCATGTGTGTCAGGATAGCCTTC	168
WNT9b	Wnt family member 9B	CTGCTCGAGTGCCAGTTTCA	CCGTCTCCTTGAAACCTCTCTT	94
VEGFA	Vascular endothelial growth factor A	ATGCGGATCAAACCTCACCA	TGTCACATCTGCAAGTACGTTCG	224
TGFB1	Transforming growth factor beta 1	GCAAGGTCCTGGCTCTGTA	TAGTACACGATGGGCAGTGG	97
TGFB2	Transforming growth factor beta 2	GCGCGATTTGCAGACTTGAG	ATGTAAAGTGGACGCAGGCA	170
REN	Renin	TCTCCGTCTACTACAGCAGGA	CCTCACAGACACCCCTTTCA	152
RET	Ret proto-oncogene	GGAAGATAACCAGGACCCGC	GTTCTTGTGGTAGCGGTGGA	124
SIX2	SIX homeobox 2	CTACCCCTCACCCCGAGAGA	TTCTCGCTGTTCTCCTCGTACC	141
CTNNB1	Catenin beta 1	ATTGAAGCTGAGGGAGCCAC	GAACTGGTCAGCTCAACCGA	152
AT1	Angiotensin II receptor type 1 (AGTR1)	GGTCTACATCCAGGTGCATT	GGGGCAGTCATCTTGGATTC	116
CDH1	E-cadherin	GCTGGACCGGGAGAGTTTTC	TGAAGATGGGTGGGTTGTCG	131
GDNF	Glial cell derived neurotrophic factor	CCTGGGCGTGTGGATGTTTA	GCCATCTGTTTATCGGGGGA	132
IGF-1	Insulin-like growth factor 1	ATTTCTTGAAGGTAAAGATGCA	CAGCCCCACAGAGGGTCTCA	117

Supplemental Figure S1 Necrotizing Enterocolitis (NEC) conditions and relative kidney weight of pigs. (a-c) NEC incidence and lesion distribution in preterm pigs. (a) NEC incidence in the preterm control and preterm IGF-1 group on PND5 and 9 (NEC defined as a score of ≥4 in at least one region). (b and c) Individual lesion scores in stomach, small intestine and colon regions on PND5 and PND9. (d) Kidney weight relative to the bodyweight. Data is presented as means ± SEM. *, $p < 0.05$, **, $p < 0.01$, ***, $p < 0.001$.

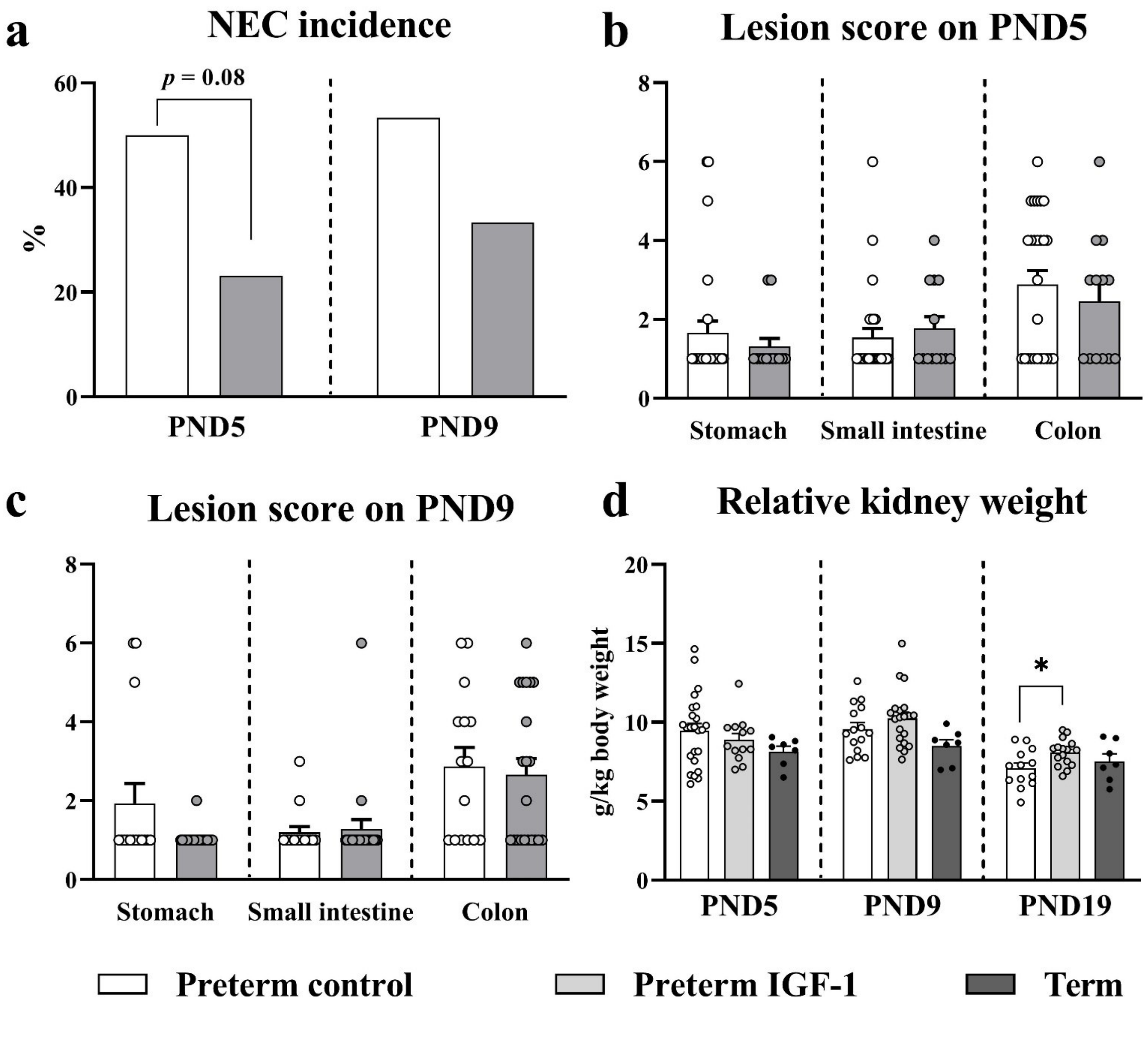

Supplemental Figure S2 The effects of NEC severity on renal parameters on postnatal day 5 and 9. (a) Relative kidney weight; (b) Urine albumin/creatinine ratio; (c) Abnormal glomeruli percentage; (d) Fractional mesangial area; (e-i) Relative expression of kidney injury and inflammation-related genes; (j and k) Kidney protein expression of TNFα and IL10; (I) Nephrogenic zone width. All data from pigs of no lesions (score 1, n=9-19), mild lesions (score 2-3, n=5-17) and severe lesions (scores 4-6, n=11-27) is presented as means ±

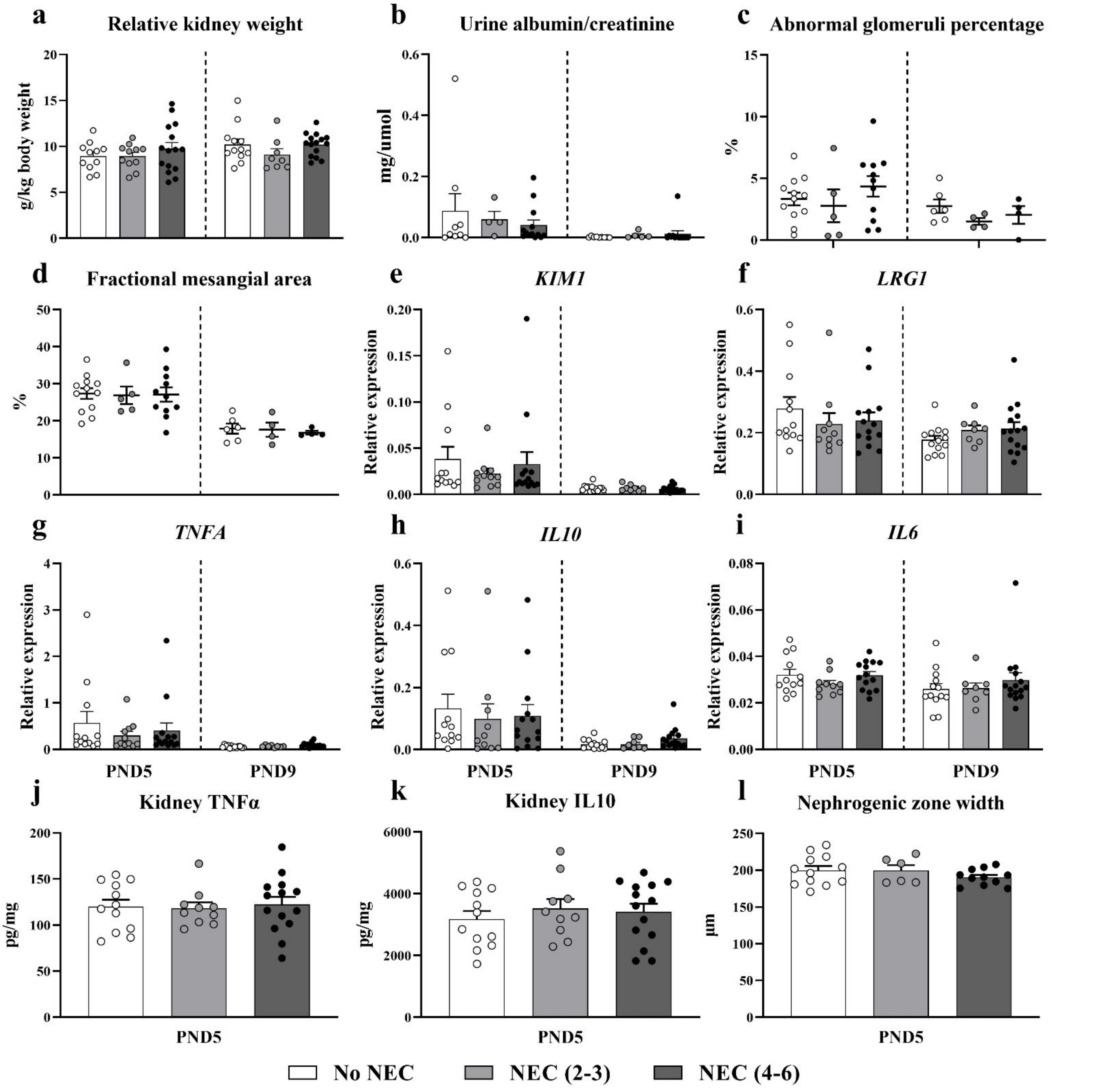

Supplemental Figure S3 Effects of NEC lesion location type on renal parameters on postnatal day 5. (a) Relative kidney weight; (b) Urine albumin/creatinine ratio; (c-g) Relative expression of kidney injury and inflammation-related genes; (i and j) Kidney protein expression of TNFα and IL10. All data from pigs of no lesions (n=20-23), non-SI lesions (n=15-32) and SI lesions (n=5-7) is presented as means ± SEM. *, $p < 0.05$, **, $p < 0.01$, ***, $p < 0.001$.

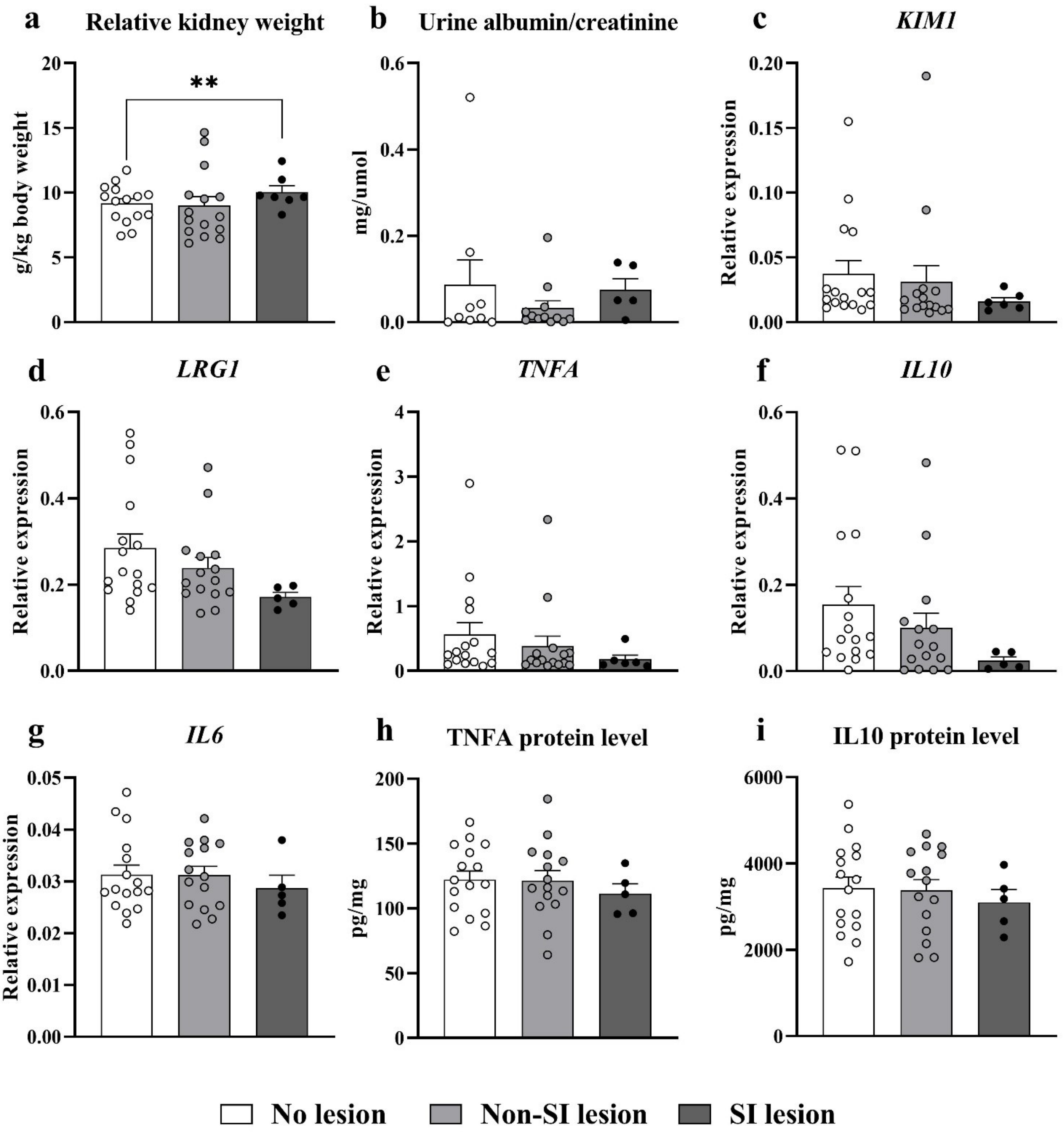

Supplemental Figure S4 Impacts of preterm birth-associated immaturity and IGF-1 supplementation on plasma and urine biochemical parameters, and estimated glomerular filtration rate during the first 19 postnatal days. (a) Plasma creatinine level; (b) Blood urea nitrogen level; (c) Plasma albumin level; (d) Urine albumin to creatinine ratio**;** (e and f) estimated glomerular filtration rates (eGFR) indexed to body weight and kidney weight, respectively. All data in preterm control (n=12-34), preterm IGF-1 (n=14-20) and term reference pigs (n=6-7) is presented as means ± SEM. *, $p < 0.05$, **, $p < 0.01$, ***, $p < 0.001$.

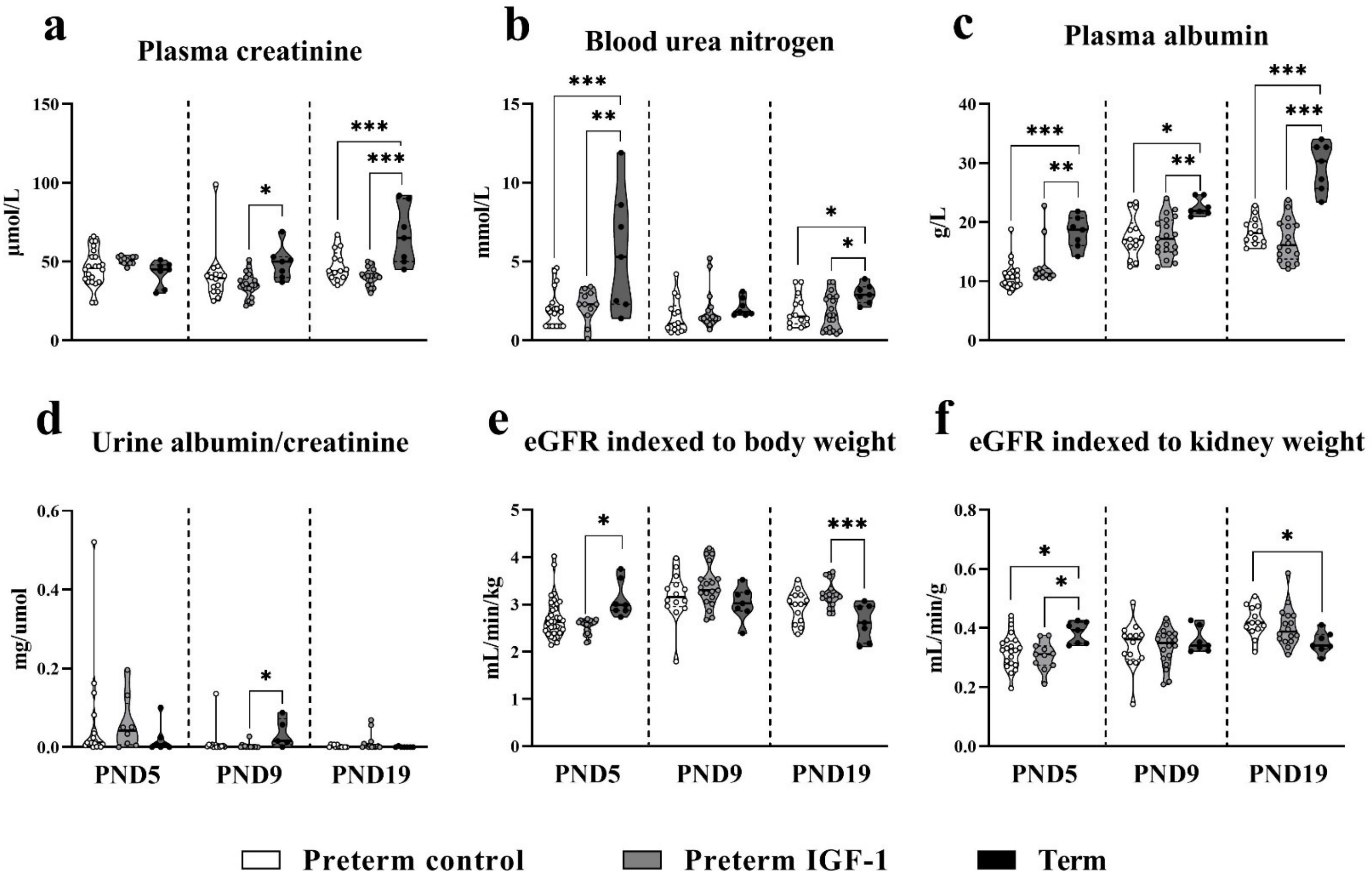

Supplemental Figure S5 Pearson correlation analysis of kidney inflammation and injury related gene expression on PND5. (a), (b), (c) and (d) are the correlation of *KIM1* to *TNFA*, *LRG1* to *TNFA*, *KIM1* to *IL10*, and *LRG1* to *IL10*, respectively.

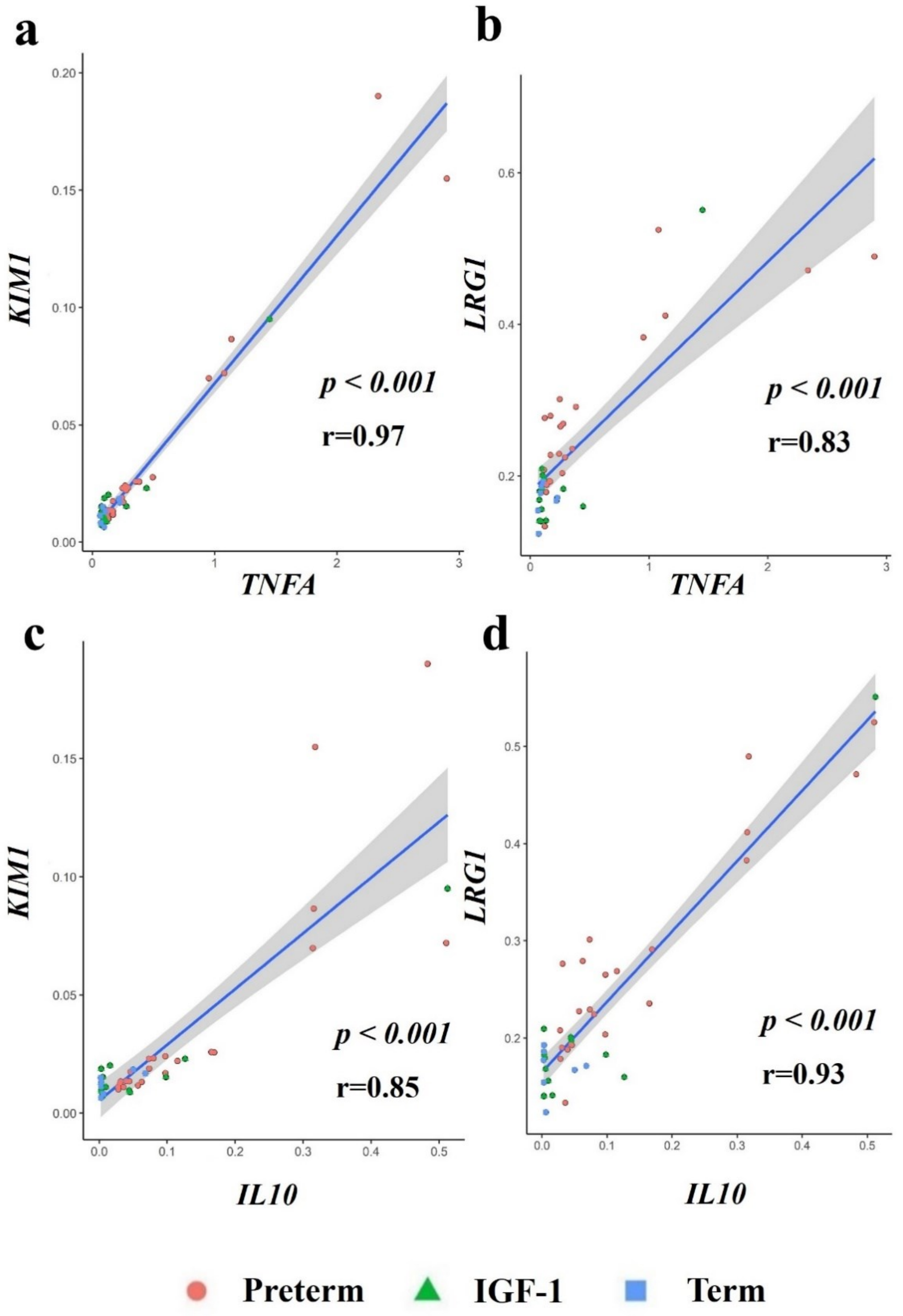

Supplement: Supplementary file 1 — Supplementary Information [file 41390_2024_3222_MOESM1_ESM.pdf]
